# Supplementary material for: SinI and SinR function differently in biofilm formation, rhizosphere colonization, and biocontrol efficacy between Bacillus velezensis and B. subtilis
Source: Microbiol Spectr. 2025 Oct 21;13(12):e02186-24. doi: 10.1128/spectrum.02186-24 (PMC12671205; doi:10.1128/spectrum.02186-24)
Supplement: Fig. S1 — Growth and sporulation of sinI and sinR knockout strains. [file spectrum.02186-24-s0001.docx]

**Figure S 1**

A


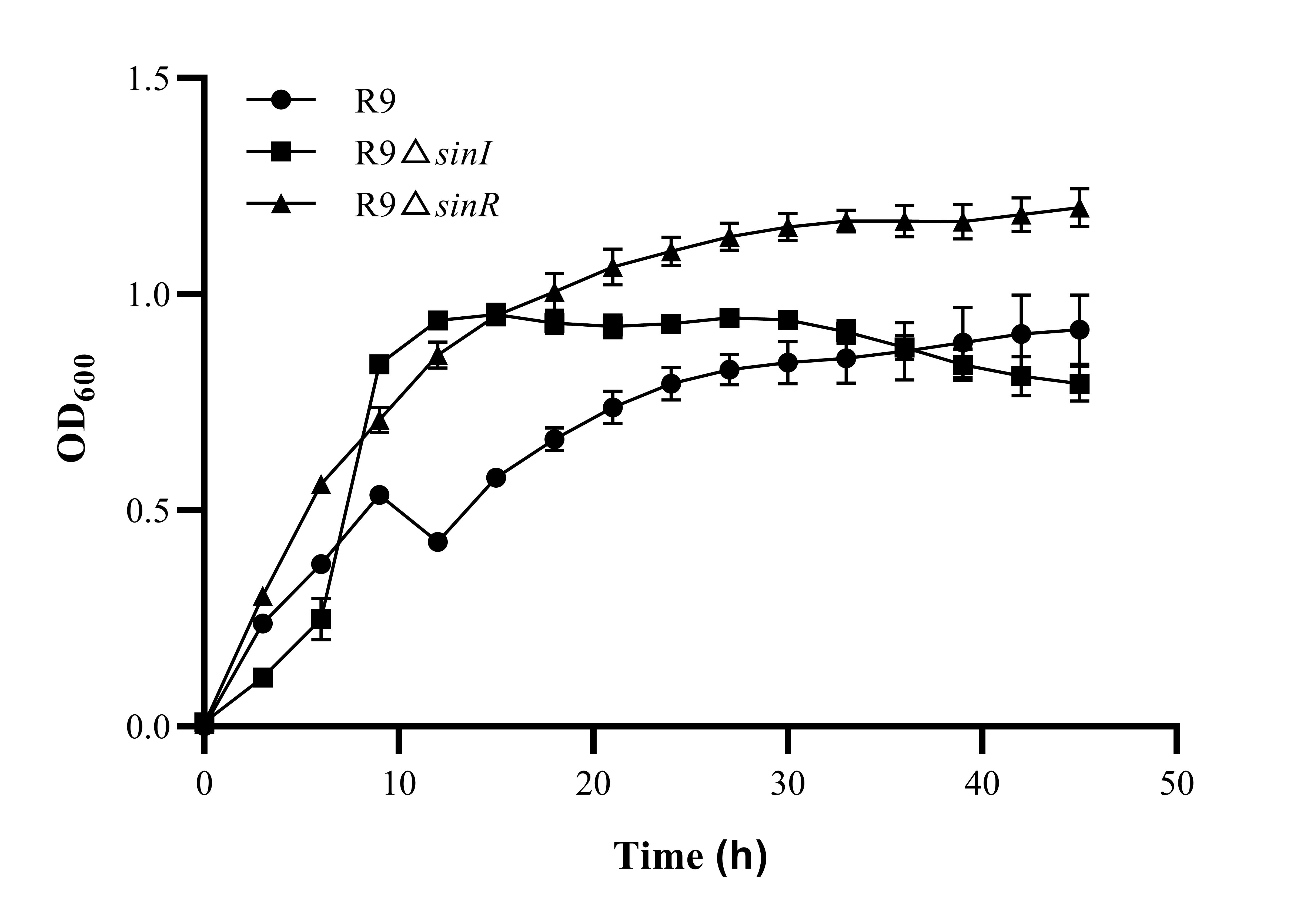


B


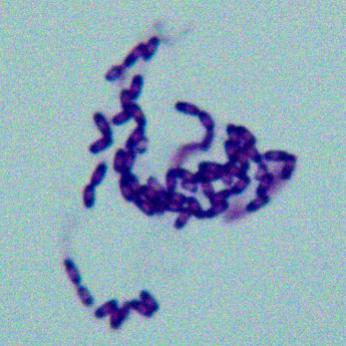


1 μm


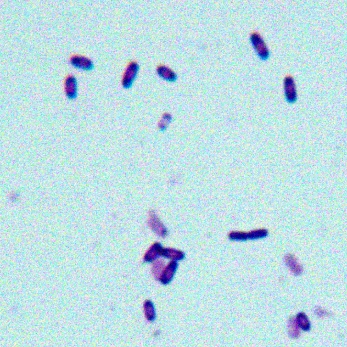


1 μm

R9

R9Δ*sinI*

R9Δ*sinR*


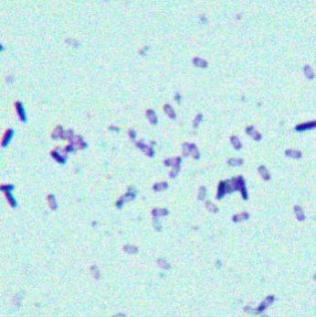


1 μm

C


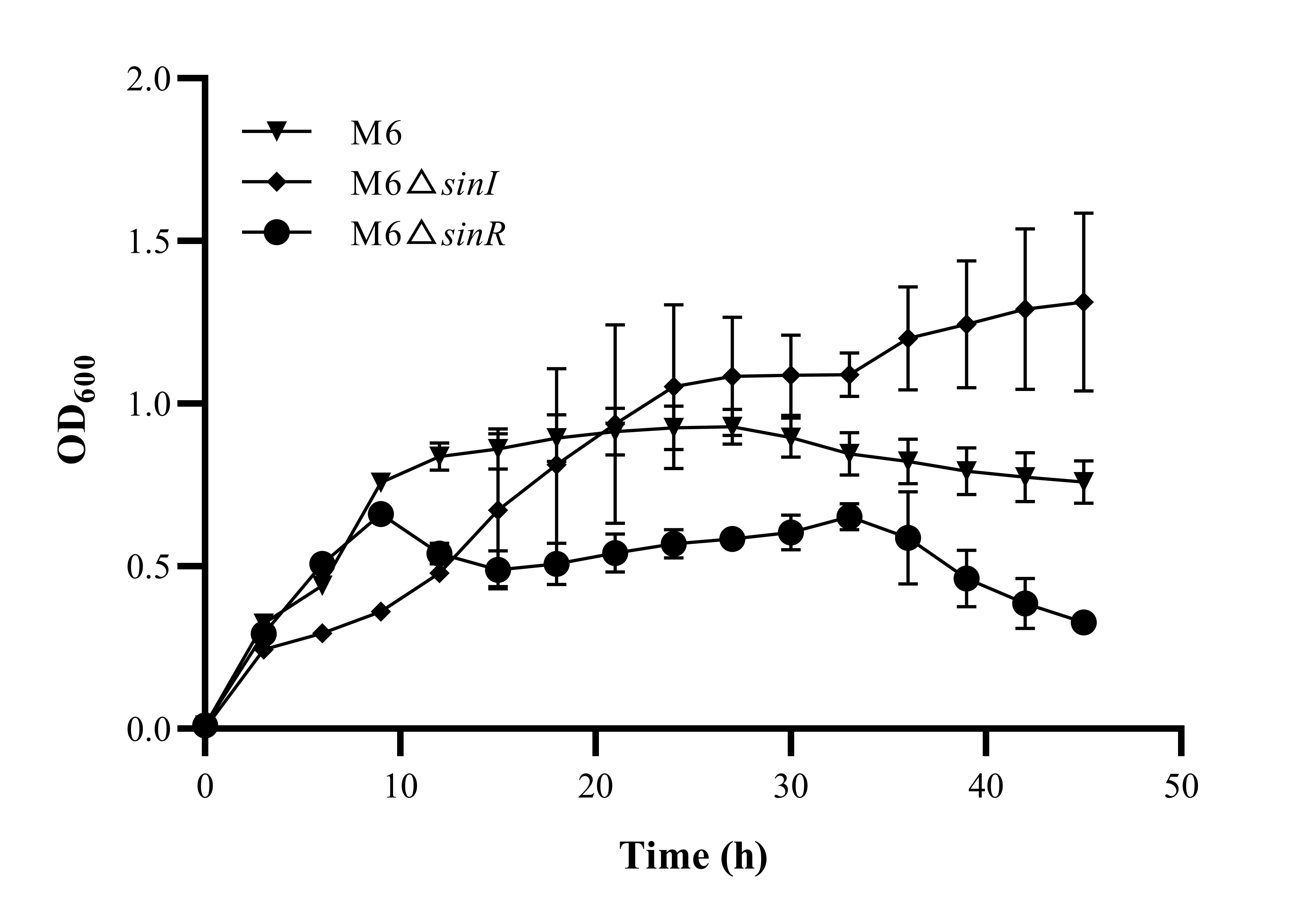


**Fig. S1 Growth and sporulation of *sinI* and *sinR* knockout strains. A:** Growth curves of R9Δ*sinI* and R9Δ*sinR*. **B:** Spores (1000 ×) of R9Δ*sinI* and R9Δ*sinR*. **C:** Growth curves of M6Δ*sinI* and M6Δ*sinR*. Error bars represent the standard deviation (SD) of the data.
