# Supplementary material for: SinI and SinR function differently in biofilm formation, rhizosphere colonization, and biocontrol efficacy between Bacillus velezensis and B. subtilis
Source: Microbiol Spectr. 2025 Oct 21;13(12):e02186-24. doi: 10.1128/spectrum.02186-24 (PMC12671205; doi:10.1128/spectrum.02186-24)
Supplement: Table S1 — Primers used in this study. [file spectrum.02186-24-s0002.docx]

**Table S1 Primers used in this study**

| Name | Sequence 5′→3′ | Purpose |
| --- | --- | --- |
| M6*sinI*-LF | C**GAGCTC**CGATTTCATACCAGTCCGAC | Amplifying L arm of *sinI* in  *B. subtilis* |
| M6*sinI*-LR | ACGGTATGACTTCTGCAGTTTCTCCTCC |  |
| M6*sinI*-RF | GGAGGAGAAACTGCAGAAGTCATACCGT | Amplifying R arm of *sinI* in  *B. subtilis* |
| M6*sinI*-RR | CG**GGATCC**GCTCCTGAATATGATGGTGT |  |
| M6*sinI*-dan-LF | GGGCCCCGCGTCTTTTTCAAATATTC | Verifying single crossover of *sinI* in *B. subtilis* |
| M6*sinI*-dan-RR | GCTAAAAATGATGCAGCGGCT |  |
| M6*sinR*-LF | C**GAGCTC**GCGGTTAACAGGTGGAAGAA | Amplifying L arm of *sinR* in  *B. subtilis* |
| M6*sinR*-LR | GCACTACTCCTCTTCGCTGGCCAATCAA |  |
| M6*sinR*-RF | TTGATTGGCCAGCGAAGAGGAGTAGTGC | Amplifying R arm of *sinR* in *B. subtilis* |
| M6*sinR*-RR | CG**GGATCC**CTCCAGAAGATTTCCTCAGC |  |
| M6*sinR*-dan-LF | TTTCAAAGCAAGCTGACCCA | Verifying single crossover of *sinR* in *B. subtilis* |
| M6*sinR*-dan-RR | ATCAAATCTAAAGCCGGGAG |  |
| R9*sinI*-LF | GC**TCTAGA**GCGTTATCCATTCGACA | Amplifying *sinI* from *B. velezensis* for expression |
| R9*sinI*-LR | ATATGGCGGCATGTCATCACCT |  |
| R9*sinI*-RF | AGGTGATGACATGCCGCCATAT |  |
| R9*sinI*-RR | CG**GGATCC**AAGTTCCAAGGCAATGCGA |  |
| R9*sinR*-LF | GC**TCTAGA**GCGTTATCCATTCGACACCT | Amplifying *sinR* from *B. velezensis* for expression |
| R9*sinR*-LR | GGCCAATCAAGCATTTTCTCCTCCT |  |
| R9*sinR*-RF | AGGAGGAGAAAATGCTTGATTGGCCAG |  |
| R9*sinR*-RR | CG**GGATCC**AAGTCGGACAGTTAAAAGAGG |  |
| M6*sinI*-LF | CG**GGATCC**CTTTTTTACCATTCGACAT | Amplifying *sinI* from *B. subtilis* for expression |
| M6*sinI*-LR | TCTGCTCAGGCATGTCATCACCTT |  |
| M6*sinI*-RF | AAGGTGATGACATGCCTGAGCAGA |  |
| M6*sinI*-RR | GC**TCTAGA**GGACCATACTGATAAAG |  |
| M6*sinR*-LF | CG**GGATCC**CTTTTTTACCATTCGACAT | Amplifying *sinR* from *B. subtilis* for expression |
| M6*sinR*-LR | CTGGCCAATCAAGCAGTTTCTCCT |  |
| M6*sinR*-RF | AGGAGAAACTGCTTGATTGGCCAG |  |
| M6*sinR*-RR | GC**TCTAGA**TAACAGCAAAAAAAAGAGACGGCC |  |

Restriction sites of *BamH* I and *Xba* I are highlighted in bold.
