# Supplementary material for: SinI and SinR function differently in biofilm formation, rhizosphere colonization, and biocontrol efficacy between Bacillus velezensis and B. subtilis
Source: Microbiol Spectr. 2025 Oct 21;13(12):e02186-24. doi: 10.1128/spectrum.02186-24 (PMC12671205; doi:10.1128/spectrum.02186-24)
Supplement: Table S2 — Primers used in qRT-PCR. [file spectrum.02186-24-s0003.docx]

**Table S2 Primers used in qRT-PCR**

| Name of gene | Sequences of primer |
| --- | --- |
| NPR1 | F: 5´-TTCATATCTCACCACCACTCTC-3´ |
|  | R: 5´-AACAGGTTCCGATGAATTGA-3´ |
| PR-1 | F: 5´-GGAGCTACGCAGAACAACTAAGA-3´ |
|  | R: 5´-CCCACGAGGATCATAGTTGCAACTGA-3´ |
| PR-5 | F: 5´-CGGTACAAGTGAAGGTGCTCGTT-3´ |
|  | R: 5´-GCCTCGTAGATGGTTACAATGTCA-3´ |
| Coi1 | F: 5´-GATTCCATCGTCCCACTTTC-3´ |
|  | R: 5´-CATAACCACCAGAGTGAGAAG-3´ |
| ETR1 | F: 5´-GGTCGCTGTGAGGGTTCCGCTTCT-3´ |
|  | R: 5´-CATGCCATTGCCTTGCACTATCTG-3´ |
| PDF1.2 | F: 5´-GGCTAAGTTTGCTTCCATCATCAC-3´ |
|  | R: 5´-CTTGTTCTCTTTGCTGCTTTCG-3´ |
| Beta-TUB 4 | F: 5´-GAGGGAGCCATTGACAACATCTT-3´ |
|  | R: 5´-GCGAACAGTTCACAGCTATGTTCA-3´ |
